# Micro-drive Array for Chronic *in vivo* Recording: Drive Fabrication

**DOI:** 10.3791/1094

**Published:** 2009-04-20

**Authors:** Fabian Kloosterman, Thomas J. Davidson, Stephen N. Gomperts, Stuart P. Layton, Gregory Hale, David P. Nguyen, Matthew A. Wilson

**Affiliations:** Picower Institute for Learning and Memory, MIT - Massachusetts Institute of Technology; Department of Brain and Cognitive Science, MIT - Massachusetts Institute of Technology

## Abstract

Chronic recording of large populations of neurons is a valuable technique for studying the function of neuronal circuits in awake behaving rats. Lightweight recording devices carrying a high density array of tetrodes allow for the simultaneous monitoring of the activity of tens to hundreds of individual neurons. Here we describe a protocol for the fabrication of a micro-drive array with twenty one independently movable micro-drives. This device has been used successfully to record from hippocampal and cortical neurons in our lab. We show how to prepare a custom designed, 3-D printed plastic base that will hold the micro-drives. We demonstrate how to construct the individual micro-drives and how to assemble the complete micro-drive array. Further preparation of the drive array for surgical implantation, such as the fabrication of tetrodes, loading of tetrodes into the drive array and gold-plating, is covered in a subsequent video article.

**Figure:**
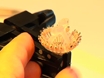


## Protocol

1. **Micro-drive array overview** A complete micro-drive array consists of several main components (Fig. 1):
  1. A computer designed plastic base that holds 21 independently movable micro-drives.
  2. A cannula at the bottom of the base in which a set of stationary tetrode guide tubes is collected.
  3. 21 micro-drives that each carry one tetrode and which can be individually driven using a screw.
  4. An electrode interface board for interconnection between tetrodes and pre-amplifiers.
  5. A protective cone (not shown in Fig. 1)
  6. In a fully assembled micro-drive array, the plastic base holds all micro-drives. Each micro-drive carries a single tetrode. The tetrode is electrically connected to the electrode interface board at the top of the micro-drive array, and passes through the stationary guide tube to exit towards the brain at the bottom of the micro-drive array. Turning the screw on each micro-drive advances or retracts the tetrode.
2. **Design of plastic base** We design the base of the micro-drive assembly in a 3-D CAD software package (SolidWorks). The advantage of this in-house design process is rapid turnover and flexibility of design. In the Wilson lab we have designed many variants of this part, geared towards the specific demands of each experiment. The 3-D model file is sent to a service bureau where it is printed from a laser-curable liquid resin using stereolithography. Turnaround time is as little as 2 days. The resulting part is lightweight, reusable, and can be machined to hold a thread. In this protocol we will use a drive base that is designed with a single recording site and which holds 21 micro-drives. The SolidWorks design file is made available upon request.
3. **Preparation of plastic base** The plastic base is processed to accommodate the micro-drives and the screws to attach the electrode interface board and the protective cone. It is not possible to reliably print smooth through holes in the plastic base, so we design undersized holes, then expand them by drilling. Carefully align the drill bit with the axis of the existing hole before drilling at very low speed. A variable speed/torque hand drill works well for this task.
  1. Drill the 21 pilot holes for the micro-drive screws (the outer ring) using a #61 bit.
  2. Drill the 21 holes for the the micro-drive support tubes (the inner ring) using a #65 bit.
  3. Drill the holes that will be used to attach the electrode interface board and protective cone using a 1.55 mm bit.
  4. Press fit a sleeve (5 mm long 20 Ga thin wall stainless steel tubing) into each of the inner ring of holes using a pair of tweezers or a pair of pliers so that it is flush with the plastic base. The sleeves ensure smooth movement of the micro-drive support tubes. Next, all holes that will hold screws are tapped. In printed plastics, thread-forming taps result in more durable threads than typical thread-cutting taps. This style of tap requires larger pilot holes. Using a hand-held pin vise to hold the tap:
  5. Tap the holes for the micro-drive screws using a tap that matches the screw size being used (M1.2x0.25) and a dry lubricant. To help align the tap with the pilot hole, insert a length of 23 Ga tubing into the sleeve next to it.
  6. Our plastic base design features a tap starter for each micro-drive screw hole to make sure the initial threads are not damaged during the threading process. After all holes are threaded, these tap starters can be easily snapped off using a razor blade.
  7. Tap the holes that will hold the screws for the electrode interface board and the protective cone using a 1-72 tap.
4. **Insertion of electrode guide tubes**
  1. To make the cannula that holds all the stationary electrode guide tubes, cut a 1.5cm-long piece of 13 Ga stainless steel tubing and roughen the surface with a dremel grinding wheel to enhance adhesion by the dental acrylic.
  2. Insert this cannula into the hole at the bottom of the plastic base, verify that it is co-linear with the vertical axis of the drive, and secure it with dental acrylic or epoxy. Leave 5 to 7 mm of the cannula exposed to facilitate surgical implantation.
  3. Cut 21 stationary electrode guide tubes (flexible polyimide tubing, inner diameter: 0.0071", outer diameter: 0.0116") with a razor blade to a length of 6  cm.
  4. Feed the guide tubes one at a time into the sleeves at the top of the drive base and all the way through the collector cannula at the bottom. Visually check for kinks or excessive bending and replace tubes if necessary. To facilitate the insertion and parallel alignment of the tetrode guide tubes, one can start with first feeding the entire bundle of 21 guide tubes up into the collector cannula from below. Then, one at a time, remove a guide tube from the bundle and feed it from the top of the plastic base through the hole that it left behind in the bundle.
  5. Push the guide tubes down until they extend only 1-2 mm from the top of the plastic base. Apply a small amount of thin cyanoacrylate glue to the tubes as they exit the collector cannula, being careful not to let any glue flow up into the end of the guide tubes.
  6. Use a fresh razor blade to cut off the excess from the bundle of guide tubes at the bottom of the collector cannula.
5. **Fabrication of micro-drives** Each micro-drive consists of a custom-machined screw and a hollow support tube (Fig. 2). The screw and support tube are attached together such that a lip on the screw is embedded in acrylic, leaving the screw to rotate freely (Fig. 2B). The screw is threaded into the plastic base, and the support tube is then driven up or down when the screw is turned, advancing or retracting the tetrode that it carries. For ease of adjustment on the behaving animal, the head of the screw is in the shape of a half-cylinder. Make a custom screwdriver for this screw:
  1. Cut 5 cm of 15 Ga stainless steel tubing.
  2. Take one of the custom screws and grind off the protruding lip.
  3. Insert the screw into the tubing until the top of the screw is flush with the end of the tubing.
  4. Crush the tubing around the screw threads repeatedly with a pair of locking pliers so that the screw cannot rotate relative to the cannula.
  5. Use a pin vise to hold the custom screw driver. To fabricate the micro-drives we use a 3-D printed plastic mold with small wells (3 mm x 5 mm, 2 mm deep). Each well has two holes in the bottom surface, spaced 2 mm apart, for the support tube and the screw. Alternatively, molds can be fabricated from an engineering plastic like Delrin, using a drill press to create the well and holes.
  6. Prepare the mold by clearing the hole for the support cannula using a 23 Ga needle and tapping the hole for the screw as described for the plastic base, above.
  7. Line the inside of the well with teflon lubricant or a thin film of petroleum jelly to allow for release of the cured dental acrylic from the mold..
  8. Apply teflon lubricant to a screw and insert it into the mold using the custom made screwdriver until the threads are just below the bottom of the well.
  9. Roughen the top 2-3 mm of a 14 mm-long support tube (23 Ga stainless steel tubing) with a  grinding wheel to enhance adhesion of the dental acrylic. Insert it into the mold until the top of the cannula is halfway between the top of the well and the top of the screw.
  10. Pour dental acrylic into the well. Agitating motion is used to ensure that the dental acrylic flows into all the spaces around the screw and support tube. Remove any air bubbles with a small needle.
  11. Wait until the dental acrylic is completely cured (15-30 minutes) and then remove the micro-drive from the mold by turning the screw counter-clock wise.
  12. It is important to do a quality check of the micro-drives. Inspect the cured dental acrylic for cracks and/or air bubbles, which are usually caused by dental acrylic that was too thick or too thin. Also, make sure the screw and the support tube are parallel to each other and that the screw turns smoothly. Screws and support tubes of micro-drives that do not meet these criteria can be cracked free from the dental acrylic and recycled.
  13. Repeat steps 5.6 through 5.11 until you have 21 micro-drives.
6. **Final assembly of micro-drive array**
  1. Lower each micro-drive into the plastic drive base. Each micro-drive support tube should move smoothly both inside its sleeve and over its stationary guide tube. Check to make sure that the guide tubes do not buckle as the micro-drive is being lowered.
  2. With the micro-drives completely lowered, add a thin layer of dental acrylic to the interior of the drive base to fix the electrode guide tubes in place.
  3. Note that failure to lower the micro drives all the way before adding the dental acrylic will result in wicking of the acrylic all the way up the sleeves and obstruction of micro-drive movement.
  4. Label the micro-drives 1-21.
  5. Turn the drive array upside down and take a picture of the bundle of guide tubes. This picture will be used to map the location of the guide tube corresponding to each micro-drive.
  6. Raise all the micro-drives by several mm.
  7. Insert a polyimide carrier tube (0.005") into each guide tube from the bottom of the drive base. Let the carrier tube extend 1-2 mm from the top of the fully lowered micro-drive and and record on the photograph the identity of the corresponding micro-drive.
  8. Glue the carrier tube to the micro-drive support tube using 5-minute epoxy or cyanoacrylate glue. Note that glue or epoxy that is too thin will flow down the support cannula and obstruct free motion of the micro-drive or, worse, fix it to the guide cannula. If this happens, that particular micro-drive unit can not be used.
  9. Fully lower all the micro-drives. Cut all the carrier tubes off flush at the bottom of the collector cannula using a fresh razor blade.
  10. Finally, mount the Neuralynx electrode interface board to the drive base using two screws (size: 1-72, length: 3/16").

At this point the drive array is ready to be loaded with tetrodes. Fabrication of tetrodes, how to load them into the micro-drives, and how to prepare the drive array for surgical implantation are the subjects of a subsequent video article.


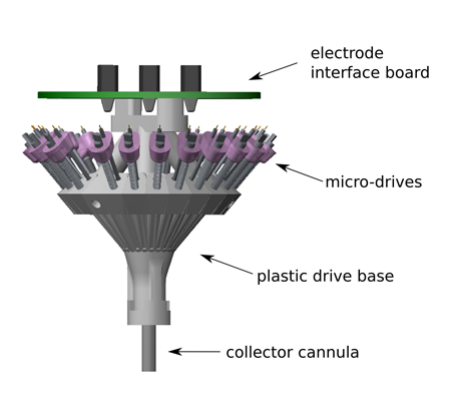
 **Figure 1.** Model of finished micro-drive array. A micro-drive array consists of several major components: a plastic drive base; a collector cannula from which the tetrodes enter the brain; 21 micro-drives that each drive a single tetrode; an electrode interface board that connects to pre-amplifiers.


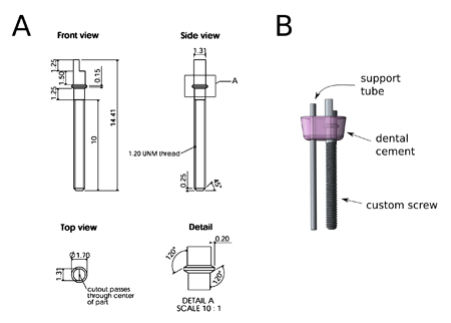
 **Figure 2.** Custom micro-drive screws. **A.** Technical drawings of the custom screw design. The screw features a threaded part (M1.2x0.25; 1.2 mm diameter, 0.25 mm / turn), a half-cylinder head that matches our custom made screw driver and a smooth part with lip (see detail). **B.** Model of complete micro-drive. The support tube and custom screw are connected with dental cement. The lip on the screw is embedded in the dental cement and allows for free rotation while driving the support tube up or down.

### Additional Files

We designed the printed drive base, the protective cone and the mold for construction of the micro-drives in SolidWorks 2008 3D CAD software:

- Download the file: DriveBase.sldprt which contains models of the drive base and protective cone and cap.
- Download the file: MicroDriveMold.sldprt which contains model of the micro-drive mold

The 3D printing service bureau can not handle native Solidworks files directly, but rather requires the design files in stereolithography CAD format (STL). For people who do not have access to Solidworks software, but still would like to use the printed parts used in the video article we include the designs in STL format as well:

- Download the file: DriveBase.stl which contains model of drive base
- Download the file: MicroDriveMold.stl which contains model of micro-drive mold
- Download the file: ProtectionCap.stl which contains model of cap for protective cone
- Download the file: ProtectionCone.stl which contains model of protective cone

## Discussion

This protocol describes the general features of the micro-drive building process. It has been successfully modified to produce drive arrays with multiple recording targets or smaller arrays for recordings in mice. In addition to recording from the hippocampus, many researchers use these drives to target cortical and sub-cortical structures. The use of longer screws may be required in order to record from deeper brain structures. With extensive modification to the drive base, motorized drives my be used in lieu of manually-turned ones, allowing more precise, remote tetrode adjustment (Yamamoto and Wilson, 2008). Customized stationary guide cannula arrangements have been used in place of the circular main cannula to achieve spatial control over the distribution of tetrodes in the cortex and hippocampus.
